# Critical-emancipatory educational intervention through games to face gender violence

**DOI:** 10.1590/0034-7167-2022-0299

**Published:** 2023-05-29

**Authors:** Lucimara Fabiana Fornari, Rosa Maria Godoy Serpa da Fonseca

**Affiliations:** I Universidade de São Paulo. São Paulo, São Paulo, Brazil

**Keywords:** Violence Against Women, Gender and Health, Education, Games Experimental, Public Policy, Violencia Contra la Mujer, Género y Salud, Educación, Juegos Experimentales, Política Pública, Violência Contra a Mulher, Gênero e Saúde, Educação, Jogos Experimentais, Política Pública

## Abstract

**Objectives:**

to analyze an educational intervention, through game “Violetas”, for the qualification of professionals who work in the fight against gender violence.

**Methods:**

a qualitative study, involving 28 professionals from intersectoral services to assist women in situations of violence, located in three Brazilian capitals. Data were collected through Critical-Emancipatory Workshops, being submitted to thematic content analysis through software.

**Results:**

sexist patterns, pornography and sexual diversity were topics listed by participants for intervention in reality. To this end, they suggested orientation actions on gender violence, promotion of individual and group assistance and service network consolidation.

**Final Considerations:**

the intervention proved to be playful, due to the use of games, critical, due to the fact that it allowed reflection on the theme, emancipatory, due to the possibility of professionals rethinking their practice and qualifying themselves to face the problem.

## RESUMO

**Objetivos:**

analisar uma intervenção educativa, por meio do jogo “Violetas”, para a qualificação de profissionais que atuam no enfrentamento da violência de gênero.

**Métodos:**

estudo de abordagem qualitativa, envolvendo 28 profissionais de serviços intersetoriais de atendimento às mulheres em situação de violência, localizados em três capitais brasileiras. Os dados foram coletados por meio de Oficinas de Trabalho Crítico-emancipatórias, sendo submetidos à análise de conteúdo temática através de *software*.

**Resultados:**

os padrões sexistas, a pornografia e a diversidade sexual foram temas elencados pelas participantes para intervenção na realidade. Para tanto, sugeriram ações de orientação sobre a violência de gênero, promoção da assistência individual e grupal e consolidação da rede de atendimento.

**Considerações Finais:**

a intervenção se mostrou lúdica, devido ao uso de jogo, crítica, pelo fato de permitir a reflexão sobre o tema, emancipatória, devido à possibilidade de as profissionais repensarem a prática e se qualificarem para o enfrentamento do problema.

## RESUMEN

**Objetivos:**

analizar una intervención educativa, a través del juego “Violetas”, para la capacitación de profesionales que trabajan en la lucha contra la violencia de género.

**Métodos:**

estudio con abordaje cualitativo, en el que participaron 28 profesionales de servicios intersectoriales de atención a mujeres en situación de violencia, ubicados en tres capitales brasileñas. Los datos fueron recolectados a través de Talleres Crítico-Emancipadores, siendo sometidos al análisis de contenido temático a través de software.

**Resultados:**

los patrones sexistas, la pornografía y la diversidad sexual fueron temas enumerados por los participantes para la intervención en la realidad. Para ello propusieron acciones de orientación en violencia de género, promoción de la atención individual y grupal y consolidación de la red de servicios.

**Consideraciones Finales:**

la intervención se mostró lúdica, por el uso de juegos, crítica, porque permite la reflexión sobre el tema, emancipadora, por la posibilidad de que los profesionales repensaran la práctica y se capaciten para enfrentar el problema.

## INTRODUCTION

Gender-based violence is a problem that interferes with women’s lives. In the new coronavirus (COVID-19) pandemic, there was an increase in the number of women in situations of violence due to the increase in the time spent in the domestic space and living with the aggressor. At the same time, there was a restriction on access to guidance and reporting channels, corroborating the aggravation of the problem^(1)^. Worldwide, one in two women reported having suffered or met someone who experienced some form of violence in the COVID-19 pandemic^(2)^. In Brazil, one in four women over the age of 16 claimed to have suffered some form of violence in the same period^(3)^.

The growth of violence against women impacts the health and economic system of countries. Research reveals that women who have suffered more than one form of violence have a longer hospitalization time and 10 times more chances of attempting suicide, when compared to those who have not experienced situations of violence. Women who experienced psychological violence report worsening in self-reported physical and mental symptoms^(4)^. There is also an increase in the cost of services provided due to the consequences of violations and the reduction in productivity of the women involved^(5)^.

Support services are essential for coping with gender-based violence, as they provide welcome and support to women. To this end, the professionals of these services need to be able to identify the problem and seek answers to users’ demands. A literature review on the effectiveness of training programs for health professionals to deal with violence against women showed that empathic and qualified care validates users’ experience and supports the understanding of their needs, which allows the achievement of better results for overcoming the problem^(6)^.

It is considered that the qualification of professionals needs to involve innovative learning methods such as educational games. A systematic review on the use of gamification in the educational context found that games positively impact on students’ learning, motivation and involvement, because the teaching process is permeated by curiosity and joy^(7)^. Another systematic review on the use of games in nursing showed the improvement of students’ knowledge and skills, as games cover realism and entertainment^(8)^.

From this perspective, game “*Violetas: Cinema & Ação*” was developed in the fight against violence against women. It is a collaborative and strategic board game. It is made up of four to eight players, who represent characters from the intersectoral network to combat violence. At the beginning of the match, each player receives a reference card that will indicate the character and their ability to action. All players move between the cities of the board for the purpose of surrounding cases of violence^(9)^.

“*Violetas*” also consists of 35 multiple-choice and essay questions, based on movie scenes that address the following themes: sexual and reproductive rights, sexual and racial diversity, gender stereotypes, forms of violence, policies, and practices for coping with violence. The questions answered correctly allow the accumulation of cards for the acquisition of tokens that guarantee victory. Meanwhile, incorrectly answered questions increase violent situations in cities and can result in game defeat^(9)^.

This study is a continuation of previous research that identified the potential of “*Violetas*” for the expansion of critical awareness about gender violence with professionals who worked in the intersectoral network. In addition to this, revealed the need for educational intervention (EI) to complement the discussions raised at the start, to deepen the understanding of the problem and the perspective of its coping^(10)^. This data was supported by another Brazilian research, which considered the game as a mediating tool in the learning process, because, in addition to encouraging dialogue and the exercise of critical analysis, it depends on the intentionality of those involved^(11)^.

Therefore, the authors of this study developed an EI proposal through “*Violetas*”, in the format of a Critical-emancipatory Work Workshop, which was the object of research with the following guiding question: how an EI, through “*Violetas*”, can qualify professionals to face gender violence?

## OBJECTIVES

To analyze an EI, through game “*Violetas*”, for the qualification of professionals who work in the fight against gender violence.

## METHODS

### Ethical aspects

The study met all the requirements proposed by Resolution 466/2012 of the Brazilian National Health Council. The research project was approved by the Research Ethics Committee of the *Universidade de São Paulo* School of Nursing, under Opinion 3,780,945/2019. It was presented to the intersectoral services that were the study setting, for knowledge and authorization request for data collection. During data collection, all necessary safety measures were respected to prevent infection by COVID-19, in order to minimize potential health risks and guarantee participant integrity.

Participant anonymity was guaranteed by replacing the name with the letter “P”, followed by an Arabic numeral. Due to the fact that participants are women, in the subsequent sections, the feminine grammatical gender will be adopted to refer to the lines.

### Study design and theoretical-methodological framework

This is an interventional study, with a qualitative approach, in which there was articulation between research and the production of knowledge for the transformation of reality through actions and processes involving the participants^(12)^. The COnsolidated criteria for REporting Qualitative research (COREQ) was used to guide the methodological path^(13)^.

It should be noted that the EI proposal was anchored in the framework of critical and emancipatory education, which considers education as a form of intervention in the world, since knowledge is constructed from community practice and the discussion about reality. Thus, the reflection process involves the movement between doing and thinking about doing^(14)^.

### Study setting

The study was carried out at the first three units of the *Casa da Mulher Brasileira* (CMB) operating in the country, located in Brasília (Federal District), Campo Grande (Mato Grosso do Sul) and Curitiba (Paraná). The CMB is the result of a public policy to combat violence against women through integrating the main specialized and multidisciplinary care network services^(15)^. The units were selected due to the time of operation in the period of data collection, with an opening date greater than five years. Thus, the intersectoral work process consolidation in the three institutions was considered.

### Data source

In this study, the number of participants was not pre-defined, as the purpose was to know the singularities and meanings associated with the phenomenon, expressed through opinions, representations, behaviors and practices^(16)^. All professionals who worked in the CMB at the time of data collection were invited to participate in the study. The participant selection process was based on convenience. Professionals who assisted women in situations of violence and who participated in a match of “*Violetas*” were included. Two professionals who did not participate in data collection were excluded.

Twenty-eight professionals working in the CMB participated in the study, five from Brasília, 14 from Campo Grande and nine from Curitiba. Data collection occurred according to participants’ availability and interest, ensuring at least four and a maximum of 16 professionals per CMB unit. This measure was adopted, because the game admits up to eight players per game, making it possible to play two games at the same time.

### Data collection and organization

Data were collected between October 2020 and October 2021, through Critical-Emancipatory Workshops (CEW). They were conducted by the first author of the study, who has consolidated experience in the data collection technique. They were also performed in the institutions themselves because they presented appropriate physical space and ease of access. The first author and the study participants were present in CEW.

CEW is a method of investigation and intervention based on critical-emancipatory education and feminist epistemology. This method is based on participation, shared responsibility, self-esteem, and empowerment. It is structured in four moments: warm-up, individual reflection, group reflection and synthesis^(17)^.

In this study, CEW was structured as an EI and an instrument for data collection. It was divided into two sessions of three hours each, performed on two consecutive days, totaling six hours per unit of CMB. The two sessions included the four moments of CEW.

At first, participants were asked about their expectations prior to the game and played a match. In the second moment, each participant chose a question from the game that attracted attention and described the repercussions of the problem for the women involved. In the third moment, the participants were divided into groups, presented the selected questions and chosen one for the elaboration of actions in response to the problem identified in the game, being applied in the study setting. Later, in plenary, the actions developed by the groups were reported and the potential and limits for implementation in professional practice were analyzed. In the fourth moment, participants selected, in magazines, images that represented the experience of each one’s participation in CEW.

### Data analysis

The data were recorded through text and audio. The texts were produced from the responses recorded on paper by participants. The audios were produced by recording the workshops, followed by full transcription, validated by the second author of this study.

Subsequently, data were submitted to thematic content analysis, which includes pre-analysis, material exploration, treatment of results, interpretation and inference, in which systematization of ideas, in-depth reading of the empirical material, categorization of speeches into themes and reflective analysis of speeches’ singularity and totality were carried out^(18)^. Material exploration and treatment of results were performed using Web Qualitative Data Analysis (webQDA)^(19)^, in order to optimize data organization and management.

In webQDA, the data was entered as internal sources. The files in DOCX format containing the speeches were divided according to each CMB. Information related to participant characterization was recorded in a file in XLSX format. The speeches were treated in the software through Tree Codes, resulting in empirical categories. Characterization was submitted to automatic import, responsible for generating the following descriptors: sex, age group, marital status, number of children, religion, skin color, education, additional training, profession, time of professional experience and time of experience at CMB.

## RESULTS

Twenty-eight female professionals participated in the study. Most participants were between 40 and 49 years old (n=10), were married (n=11), had one or more children (n=19), reported being Catholic (n=14) and declared themselves white skin color (n=17). All had completed higher education, and most reported having at least one graduate course (n=19). With regard to work, they worked as psychologists (n=11), social workers (n=10) and administrative technicians (n=7), with more than 10 years of experience (n=18) and for more than four years in the CMB (n=18).

Data analysis allowed the emergence of three empirical categories: *Situations that demand professional performance; Actions that support the fight against gender violence; Potential and limits for implementing actions in reality*.

### Situations that demand professional performance

“*Violetas*” was used as a mediating tool for the production of knowledge about gender violence through a theoretical approach (questions) and practice (collaborative dynamics). The experience of the match motivated the reflections built in EI. Based on the gender issues discussed by participants in the game, three guidelines were listed, which require greater investment in the context of CMB’s operations: the deconstruction of sexist patterns, the risks of pornography and respect for sexual diversity.

Participants discussed the standards of femininity expected for women in society, understanding that such standards reinforce female subalternity in relation to male ones, support the idea of being perfect and regulate situations that aim to curb any propensity to disobedience to the patriarchal social order.

*Women are forced to fit into a social pattern in which women must meet all the expectations and demands of men, losing their identity. They do not accept to fit into this reality and start to pursue, seek a way out, until discovering that there was a manipulation over women so that they were all “perfect” in the eyes of men.* (P16)

The participants reflected on the impact of pornography on women’s lives, to what extent it is a source of pleasure or violence, drawing attention to the naturalization of forced sexual intercourse and the sending of images containing the naked body to partners.

*People end up watching porn movies and taking those practices into their personal lives, as if that were the right thing, the right thing, which is what they want, but is it? Is that what I really want? Is this sexual practice the one I want or is it the one that was imposed, or is it the one that my partner who is addicted to pornography wants me to submit to?* (P25)

According to participants, sexual diversity was chosen because of the challenge implicit in the reflection on the topic. This aspect was evidenced in professionals’ workspace and private lives. Some reported family conflicts due to the sexual orientation of one of their members.

*As a mother, I idealized my daughters: marriage, grandchildren and all. My youngest daughter, a year and two months ago, decided to tell me, she gave me a daughter-in-law as a gift. I had to do the deconstruction work to accept it, I used the term for a long time: “look, I respect your decision, but I don’t accept it”.* (P14)

In the three agendas, participants commented on the difficulties of women to break their affective relationships with the occurrence of situations of violence. Recognition of the problem was considered the first step of a long journey towards overcoming.

*Women in marriage have to know how to deal with the men they married, marriage doesn’t break, it’s for life* […]. *So, there are all these beliefs that they feed, it’s not enough to be comfortable, but I think it’s blindness, a lack of perception about her condition within the relationship.* (P4)

To overcome the problem, professionals mentioned the need to encourage individual empowerment and women’s autonomy so that they become active in the coping process. They also commented on the participation of men in the deconstruction of dominant power relations, overcoming the exclusive role of defendant.

*To what extent do women in society, in different contexts, have the power of choice? I think that’s what I had to work a lot with women, with society in general, but with women too, for them to believe that they have the power to choose. They must have autonomy, not wait.* (P9)

*The aggressor must also be involved in the process. I don’t think we will be able to make much progress, because what he does today with Maria, with Joana, with Eva, if nothing is done, he will do with someone else* […]. *Of course, we are attending to the woman, but we also have to think that there is another person involved, which is also the result of an extremely sexist upbringing.* (P21)

### Actions that support the fight against gender violence

Through the guidelines raised by “*Violetas*”, participants proposed interventions in reality. Initially, they expressed difficulty in indicating actions to overcome a problem, whose complexity comes from its origin in the structure of society. Subsequently, they discussed and assessed the actions implemented in practice related to coping with violence. Finally, they developed actions in response to the game’s questions that are evidenced in the study settings.

The dissemination of the theme to the community through campaigns, lectures and booklets was widely cited. According to participants, there should be more campaigns in order to increase the number of complaints. The booklets were considered a relevant way to guide women, and the lectures were pointed out as a way to prevent the problem, especially when carried out with adolescents and young people.

*The increase in the number of complaints is a consequence, for example, of campaigns. August is the month of lilac, so the number goes up there.* (P9)

*I think it’s a guide to aid women in the legal process, so they know the steps, the terms they sometimes don’t understand. A booklet informing you about court proceedings and her rights.* (P5)

*Let’s talk to this new generation to prepare them to gradually change this social structure, so that this doesn’t happen again. There are people who can talk, this is utopia! I prefer to think that, in a few years, this will not happen again.* (P18)

Moreover, participants mentioned the offer of individual assistance to women. They proposed that, in the first meeting, there should be reception and positive reinforcement so that she feels supported and establishes a relationship of trust with professionals.

*According to what we put here, as an expression of psychology, positive reinforcement, which is nothing more than talking about what is good in her, that she starts to discuss only the skills, what she has as a person, as woman, even as a professional, or as a housewife.* (P23)

Through the bond of trust established between professional-user, participants suggested the inclusion of women in care groups, as they constitute spaces for sharing experiences and social support. In these groups, the woman may have contact with other similar life stories.

*What I really like about this group issue is that they go through the same situations. So, her hearing from a woman who is going through the same thing, I think it enriches her a lot. When she hears from us, “ah, you’re in a comfortable situation, you don’t know what violence is”. So, I think that even her own appreciation, of hearing from someone else’s mouth, I think that enriches her a lot.* (P24)

In addition to the above, participants mentioned actions to face violence that were not directly associated with women. They reflected on the need to qualify professionals from the moment they enter the care services, regardless of their area of activity. This measure was justified by the fact that the topic is still little addressed during university education.

*We had massive training when we took office here at CMB. It was more than a week, every day, the whole working day, with professionals talking about gender, about violence, Maria da Penha herself was here* [...]. *So, this was very important, because we were arriving, we were new. I had no knowledge, no experience in relation to domestic violence, so it really gave us a very solid foundation for us to start working.* (P18)

The qualification of professionals was considered essential for the implementation of care protocols and the establishment of effective intersectoral work. To this end, participants suggested carrying out case studies aimed at joint discussion of alternatives to face the peculiar situations of the women assisted.

*It’s as if we took a case, every day we took a case and we’re going to discuss, let’s see if I was attending to Dona Maria, what my role would be. We would more or less suggest, but wouldn’t we have alternatives that were not thought of? This I really think would help because every day is the most different situations.* (P21)

### Potential and limits for implementing actions in reality

Then, in action elaboration, participants reflected on the implementation process in reality. From this perspective, they discussed women’s access to CMB services. They recognized that the COVID-19 pandemic interfered with the decrease in the offer of face-to-face services. They also questioned the possible causes of the increase in the number of women assisted, as they may indicate the growth of cases of violence or greater demand, due to access to information. This aspect was pointed out as an assessment of the actions developed.

*To what extent am I really able to assist everyone who is going through this situation of violence? Because we know that most who go through the situation do not seek the service. So, it is very difficult to quantify, are we going to quantify, to assess why there was more demand from women during these months? This is positive on the one hand, but on the other hand is there more violence?* (P9)

There was recognition that the fragmentation of multidisciplinary care weakens violence coping. The need to talk repeatedly about the situation experienced by professionals from different sectors can result in the availability of inconsistent information that interferes with the judicial process. In view of this, they reported concern about the discontinuity of care given to women, as there is no return on the results of referrals.

*Another thing, I feel this immensely, I don’t know what will happen to this woman when I hand her over to the police station, if they accepted the police report, if she received the protective measure, if she went to the services that we referred.* (P22)

When there is a return of care, they do not always reflect actions that effectively supported women. On the contrary, they sometimes hindered the process of overcoming the problem even more. This aspect was mainly associated with the public security and justice sectors, since, by reproducing the patriarchal system, they blame women for situations of violence.

*I think justice is a bit masculine, if we stop to think about it, and this is sometimes not even said, it’s implicit, I think there’s this discouragement. They go to the police station, they suffer another questioning, but what did you do? What was your part? So, they stop being victims and end up leaving.* (P2)

On the other hand, there was the sharing of successful experiences developed at the CMB, which can be added to the actions developed from “*Violetas*”. The individual online service was highlighted, which resulted in maintenance of urgent protective measures, the integrated case studies, involving the service sectors to guide women, and follow-up until violence was overcome.

*The telecall service broke the objection, because sometimes you call the woman, especially those who have already passed by and are revoking the measure, if you invite, if you call for her to come, she doesn’t come, but if you make a call video via WhatsApp, she answers.* (P7)

*We thought, mainly, about the case studies strategy, also thinking about what we lived in the CMB, in the integrated studies, along with the other bodies, and the ease - I’ll put it in quotes - because it wasn’t perfect, but it was a way that we actually had to make women have access to other policies, and we understood that there was agility in the guidelines, at least, or even in the course of the processes.* (P1)

*After we finish this service with her, she warns that it is ending considering her emancipation, her insertion in the job market, that she no longer suffers violence, that the measures are being respected, she informs that the CMB works 24 hours and will be always at your disposal, but for now we will close the case.* (P10)

In addition to participating in the workshop providing this type of reflection on the practice and the actions that need to be implemented, it allowed the discussion of gender issues and the challenges for facing violence in reality, mediated by the game.

*Because you have a thousand things to discuss on this topic, and that’s cool too, having this space for discussion for reflection.* (P25)

Participants positively assessed the way EI was systematized, because, despite dealing with a difficult topic for women, both users and professionals, promoted the strengthening and inspiration for the elaboration and implementation of collaborative actions aimed at combating gender violence and constitute advances in public policies to guarantee women’s rights.

*I think I left here a little more inspired, because I saw myself strengthened with the girls, our thinking together. We are going through a dismantling of politics, more specific to women, and seeing our thoughts converging inspired me.* (P2)

## DISCUSSION

The study is innovative, as it presents an EI proposal for the qualification of professionals who work in the intersectoral network. The results showed that “*Violetas*” promoted the involvement of professionals, the sharing of perceptions on gender issues and reflection on coping with gender violence, in a pleasant and playful way, despite the difficulty of dealing with the topic.

This data corroborates the results of a systematic review on the use and impact of game-based learning in nursing, in which students’ experience was considered pleasant, engaging, and motivating. It also improved teamwork and student relationships^(20)^. In addition, another systematic review on the use of gamification in health professionals’ education showed progress in outcomes, behaviors and attitudes related to learning^(21)^.

“*Violetas*”, added to the EI, provided the discussion about aspects that enhance or hinder the work of psychologists, social workers, and administrative technicians in facing gender violence in a horizontal, participatory, and collaborative way. Furthermore, it encouraged the suggestion of actions in response to problems in professional practice. In summary, it was observed that the creation of a playful experience to question reality contributes to the transformation of care for women in situations of violence in the study settings.

Gender violence is a social phenomenon that impacts men’s and women’s lives and has different expressions in society. According to participants, recognizing the problem is the first step towards coping, because, in most cases, there is no understanding that the lived experience is about violence. This directly influences the extension of time for the request for help and the interruption of the judicial process against the aggressors.

An integrative review on the factors that influence the silencing of women in situations of violence identified the concern with the image of the family and children, the consideration that it is a private matter, the feeling of self-blame, fear of the aggressor and social reactions, emotional and financial dependence. Moreover, it highlighted the concern about inadequate responses from health and social care professionals, expectations about gender roles, the naturalization of violence and conservative religious values^(22)^.

With regard to the responses of health professionals, the literature has different perspectives, depending on the social context in which women are inserted. In this regard, an African survey of 23 Primary Health Care participants identified the responsibility of users for violence, due to passive behavior or alcohol consumption^(23)^. In contrast, a Canadian survey of 30 women linked to shelters found non-passivity because they resisted violence and acted to preserve autonomy. For a portion of them, the permanence in an abusive relationship was understood as the best or only available option^(24)^.

The promotion of women’s economic and emotional autonomy was interpreted by the study participants as synonymous with becoming participatory in the coping process, especially for decision-making. Brazilian research, with 16 women who denounced their aggressors, considers that women’s autonomy should be encouraged from childhood. For that, it is necessary a socialization that problematizes gender inequalities and promotes a critical understanding of sexist patterns, since men and women are subject to culturally and historically constructed gender norms^(25)^.

Although the CMB service is centered on users’ needs, the study participants discussed the approach of aggressors beyond the role of defendant in the judicial process. They considered that punishment is not enough to change sexist behaviors and that it is necessary to offer specialized care that promotes critical reflection on gender issues that constitute the substrate of situations of violence.

A survey carried out in Jordan, with 14 men, on the perception of violence against women, revealed that male domination was recognized by a portion of participants as a way of assuring men control over women’s lives. Violence was understood as a way of resolving family conflicts and disciplining bad behavior that disfavors male power. Participants suggested the education and counseling of men as actions to face the problem^(26)^. A systematic review on assessing interventions to reduce violence against women and girls in Sub-Saharan Africa found that including men in community workshops on changing beliefs and gender norms made it possible to reduce violence^(27)^.

Based on “*Violetas*” questions and reality, the participants of this study listed actions aimed at women and specialized services. Participation in the game raised reflections that allowed the construction of actions, in a collaborative and participatory way, with a view to professional practice involving guidance on gender violence, the promotion of individual and group assistance and the service network consolidation.

Guidance on gender-based violence was evidenced in research related to the implementation of interventions to reduce the problem in Central Asia, South Asia, and Sub-Saharan Africa. The study highlighted that interventions need to be designed based on the local context, target the factors that drive violence, offer comprehensive support to women in situations of violence, involve men and women, and allow for in-depth reflection on gender issues. It also suggested that the implementation be carried out through group activities with positive interpersonal relationships and participatory methods^(28)^.

The potential of group activity to face violence punctuated by the participants of this study was elucidated in Brazilian research with 29 women and nine professionals from two Justices of the Peace. It found that women’s groups favor female reflection and empowerment, because they provide support, care, and self-knowledge. Moreover, they make it possible to express feelings, share experiences and rethink behaviors^(29)^.

The participants of this study reflected that coping with violence does not depend exclusively on the woman involved but requires a service network as a response to the consequences of violence and the consolidation of public policies to promote gender equality in different dimensions of society. Regarding the service network, participants considered that the offer of specialized services does not guarantee overcoming the problem, since, in practice, the fragmentation and discontinuity of care to users are perceived.

This result is reported in a Brazilian study with 30 professionals from a multidisciplinary team responsible for the care of women who experienced sexual violence. As limits, problems related to the flow of care, lack of instrumentalization of professionals, revictimization, communication failures and access to information, poor physical structure, human resources deficit and care protocols were pointed out^(30)^.

Participants reported that the service network fragility influences the discontinuity of coping with violence. As a barrier to implementing actions in professional practice, the care provided to women in situations of violence, by the public security and justice sectors, in all study settings was highlighted.

Brazilian research with women and professionals from two Courts of Justice assessed that the police and legal assistance is a resource that supports women to face violence. However, the long period and the different levels of judgment may lead to withdrawing the report. Professionals in the service network are responsible for guiding women to encourage continuity of the judicial process^(29)^.

In addition to the legal process being onerous, participants reported that women do not always receive adequate reception and listening. This data corroborates the results of a systematic review on police attitudes in the intervention of violence against women. It was found that 61.4% of articles reviewed addressed police intolerance, with emphasis on blaming the victim, exempting the aggressor, reproducing gender stereotypes, and accepting violence. This tolerance reflected in the passivity of professionals during the intervention^(31)^.

The absence of professionals’ support for interventions to combat violence was also observed in North American research, which analyzed the implementation of an intervention in the social support network. The results revealed organizational capacity, concern for the safety of women in situations of violence and difficulty in care for aggressors^(32)^.

The qualification of professionals for the service was seen as a possibility to overcome the limits mentioned by this study participants. In this regard, a survey of 377 students in the field of health and social assistance from five countries highlighted that most participants had not received guidance on an adequate approach to gender-based violence in university education, therefore, they did not feel prepared to intervene in situations. They also valued practical-based learning when compared to strictly theoretical discussions^(33)^.

A systematic review on educational strategies to address violence with health students identified lectures or seminars, tutorials, interactions with users, peer education, theater, and interactive workshop as interventions. It revealed that interactive learning strategies had better results than didactic activities with passive participation^(34)^.

Interactive learning focused on practice was highlighted by the participants as a positive aspect of the EI proposed in this study. The workshop was characterized as an important moment for discussion about CMB’s daily situations because the routine of care, marked by the shortage of professionals and work overload, it does not always allow pauses to reflect on what is being implemented and how it effectively contributes to coping with gender violence.

A systematic review on interventions addressing gender stereotypes and norms found that the proposals for participant education, involving men and women, during several sessions, had greater success for behavior transformation. The results also draw attention to the construction of a welcoming environment and positive relationships, allowing further deepening on the content^(35)^.

Another study, related to qualitative assessment of four programs to prevent violence against women in Ghana, Rwanda, South Africa and Tajikistan, found that group educational interventions, which promote the sharing of experiences and critical reflection on gender, were better assessed, as they enabled the production of new ideas, the transformation of behaviors, the strengthening of relationships and trust between those involved^(36)^.

### Study limitations

The study was limited by the small number of professionals who participated in data collection, due to the COVID-19 pandemic and the restriction measures applied in each setting. There was also the absence of health professionals who, despite being one of the characters in the game, are not part of the sectors that make up the CMB, indicating the need to expand the study to include health services.

### Contributions to health and public health policies

The results revealed that the EI proposal through games has the possibility to encourage reflection on gender, as well as the implementation of actions aimed at transforming participants’ reality in terms of coping with gender violence. Thus, the results contribute to the implementation of Brazilian public policies for the defense of women’s rights, for the qualification of students and professionals from different areas of knowledge involved in the care and support for women at risk or in situations of violence.

## FINAL CONSIDERATIONS

Participants considered that the EI proposal through games is powerful to encourage collaborative learning and the qualification of professionals who work in the fight against gender violence in the study settings. EI was also playful, due to the use of board games, critical, for allowing reflection on the expressions of gender violence implicit in reality, and emancipatory, for allowing (re)thinking assistance to women and the practices developed in the services that make up the care network.

Additionally, it is considered that, given the Brazilian context of dismantling, and underfunding of public policies in defense of women’s rights, in addition to the qualification of professionals, EI makes it possible to strengthen the intersectoral network, because it aims to implement actions in reality that go beyond the fictitious experience of the game and positively transform the process of facing gender violence for women, professionals and care services.

## Supplementary Material

0034-7167-reben-76-s2-e20220299-suppl01

## Data Availability

https://doi.org/10.48321/D1MC70
