# Supplementary material for: Critical-emancipatory educational intervention through games to face gender violence
Source: Rev Bras Enferm. 2023 May 29;76(Suppl 2):e20220299. doi: 10.1590/0034-7167-2022-0299 (PMC10226417; doi:10.1590/0034-7167-2022-0299)
Supplement: 0034-7167-reben-76-s2-e20220299-suppl01 [file 0034-7167-reben-76-s2-e20220299-suppl01.pdf]

Plan Overview

A Data Management Plan created using DMPTool

DMP ID: https://doi.org/10.48321/D1MC70

Title: Potencialidades e limites do jogo Violetas para o enfrentamento da violência de gênero

Creator: Rosa Fonseca - **ORCID:** 0000-0001-9440-0870

Affiliation: Universidade de São Paulo (www5.usp.br)

Data Manager: Lucimara Fabiana Fornari

Contributor: Maria Raquel Gomes Maia Pires, Rebeca Nunes Guedes de Oliveira

Funder: São Paulo Research Foundation (fapesp.br)

Funding opportunity number: 2017/11960-6

Template: Template USP - Mínimo

Project abstract:

A violência de gênero é um fenômeno social, complexo e historicamente construído, responsável por provocar danos psicológicos, físicos e sociais à qualidade de vida de quem a vivência. Requer dos profissionais que atuam nos serviços de atendimento as mulheres em situação de violência um conhecimento generificado. Para tanto, se faz necessária a utilização de tecnologias, ferramentas e estratégias educativas inovadoras que estimulam a compreensão da violência de gênero. Nesta perspectiva, o jogo Violetas mostra-se como uma tecnologia educativa que possibilita aos jogadores uma aproximação com essa temática. O objetivo dessa pesquisa foi compreender as potencialidades e os limites do jogo Violetas para o enfrentamento da violência de gênero. Tratou-se de um estudo exploratório e descritivo, de abordagem qualitativa. A aproximação e a compreensão do objeto de pesquisa foram realizadas a partir das categorias analíticas de gênero, violência de gênero e educação crítico-emancipatória. A pesquisa teve como

cenário de investigação as unidades em funcionamento da Casa da Mulher Brasileira, localizadas em Brasília, Campo Grande e Curitiba. Os participantes da pesquisa foram os profissionais dessas instituições, responsáveis ou envolvidos no atendimento de mulheres em situação de violência. Os dados foram coletados a partir de Oficina de Trabalho Crítico-emancipatória e analisados segundo a proposta de Bardin para a emergência de categorias empíricas, com o apoio do software WebQDA. O estudo foi aprovado pelo Comitê de Ética em Pesquisa da Escola de Enfermagem da Universidade de São Paulo. Com este estudo pretendeu-se a compreensão do potencial do jogo Violetas na ampliação da consciência crítica dos profissionais para o enfrentamento da violência de gênero.

Start date: 02-28-2018

End date: 02-27-2022

Last modified: 11-25-2021

Copyright information:

The above plan creator(s) have agreed that others may use as much of the text of this plan as they would like in their own plans, and customize it as necessary. You do not need to credit the creator(s) as the source of the language used, but using any of the plan's text does not imply that the creator(s) endorse, or have any relationship to, your project or proposal

---

Potencialidades e limites do jogo Violetas para o enfrentamento da violência de gênero - Descrição dos Dados e Metadados produzidos pelo projeto

Descrição dos dados e metadados produzidos

Que dados serão coletados ou criados?

Os dados foram coletados por meio de Oficinas de Trabalho Crítico-emancipatórias.

As Oficinas de Trabalho foram registradas por meio de áudio no formato .MP3 e por meio de vídeo no formato .mov.

Os documentos preenchidos pelos participantes no decorrer das Oficinas de Trabalho foram transcritos para o formato de documento digital .doc e salvos no formato .pdf.

Os discursos das Oficinas de Trabalho foram transcritos na íntegra para o formato de documento digital .doc e salvos no formato .pdf.

As notas de observação participante foram escritas manualmente em um caderno de registros e transcritas na íntegra para o formado de documento digital .doc e salvas no formato .pdf.

Todas as fontes de dados salvas no formato .pdf foram utilizadas para tratamento e análise no *software* de análise qualitativa webQDA. Neste software, o armazenamento é vitalício, somente a licença para edição é temporária.

No total foram coletados: 18 horas de vídeo e 18 horas de áudio. As 18 horas de áudio transcritas na íntegra para o formato de documento digital resultaram em 400 páginas. Além disso, foram registradas 60 páginas de notas de campo.

Como os dados serão coletados ou criados

A coleta de dados foi realizada por meio de Oficina de Trabalho Crítico-emancipatória com duas sessões de três horas de duração cada. Além disso, foi realizada observação participante nos cenários de pesquisa com duração de cinco dias. A pesquisa contou com três cenários de pesquisa, situados em Brasília (Distrito Federal), Campo Grande (Mato Grosso do Sul) e Curitiba (Paraná).
